# Manufacturing and Recycling Impact on Environmental Life Cycle Assessment of Innovative Wind Power Plant Part 1/2

**DOI:** 10.3390/ma14010220

**Published:** 2021-01-05

**Authors:** Krzysztof Doerffer, Patrycja Bałdowska-Witos, Michał Pysz, Piotr Doerffer, Andrzej Tomporowski

**Affiliations:** 1 Department of Manufacturing and Production Engineering, Faculty of Mechanical Engineering, Gdansk University of Technology, 80-233 Gdansk, Poland; 2 Department of Technical Systems Engineering, Faculty of Mechanical Engineering, University of Science and Technology in Bydgoszcz, 85-796 Bydgoszcz, Poland; patrycja.baldowska-witos@utp.edu.pl (P.B.-W.); a.tomporowski@utp.edu.pl (A.T.); 3 Department of Energy and Industrial Apparatus, Faculty of Mechanical Engineering, Gdansk University of Technology, 80-233 Gdansk, Poland; michal.pysz@pg.edu.pl; 4 Centre of Flow and Combustion, 80-231 Gdansk, The Szewalski Institute of Fluid-Flow Machinery Polish Academy of Sciences, 80-231 Gdansk, Poland; doerffer@imp.gda.pl

**Keywords:** innovative wind turbine, Eco-indicator 99, LCA, recycling, wind energy

## Abstract

Wind power plants are considered as an ecologically-clean source of energy. However, manufacturing processes cannot be treated that way. Manufacturing processes consume huge amounts of electrical and thermal energy and significant amount of materials, e.g., steel, polymers, oils, and lubricants. All of the above could be potentially harmful for environment. There are not many works and publications regarding life-cycle analysis of wind power plants. This study’s objective is to use LCA (Life Cycle Assessment) to the manufacturing and utilization of a specific drag force-driven wind turbine. The discussed innovative wind turbine is of the type that assures safety for prosumer application. Drag force-driven turbines become more heavy than other types of lift driven turbines, but at the same time, their characteristic provides opportunity to use easily recyclable materials instead of materials like plastics or composites. The wider look through LCA tools, may change the perspective of view at that type of wind turbines. Analyzed turbine has capacity of 15 kW and is located in Poland. LCA was carried out using Eco-indicator 99 method in eleven impact categories. Among all of the turbine components, the highest negative impact was noted in the case of the tower. The wind turbine under consideration is characterized by high recycling potential. According to the presented research, recycling provides around 30% reduction of the environmental impact.

## 1. Introduction

Wind is an important renewable source of energy. The advancement of wind energy power plants leads to the reduction of conventional fuels demand (hard coal, oil, natural gas), whose resources constantly deplete due to industrial development. 

Extensive industrial development and advancing energetic resources consumption lead to progressive degeneration of ecosystem and natural landscape [1]. Improvement of technologies based on alternative sources of energy is vital. Wind energy is one such alternative source. Wind power plants produce “green” energy without hazardous emissions. However, there is no such technology that will be fully harmless for the environment [1,2]. The development of integrated usage scheme of wind power plants—their designing, manufacturing, exploitation, and their post-utility management could possibly minimize environmental and human health harmfulness.

**Figure 1:**
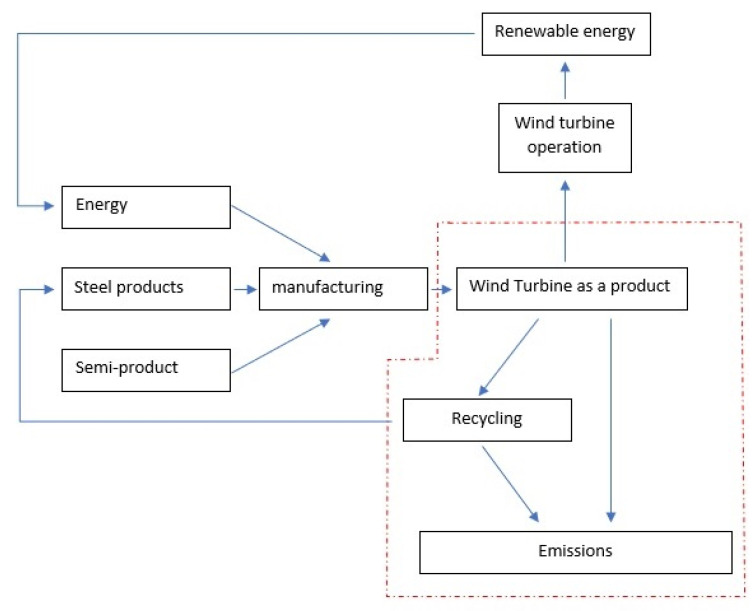
Flow diagram of used methodology with system boundaries.

In order to estimate the influence of wind power plants on the environment, the LCA (Life Cycle Assessment) method is used. This study contains two life cycle phases: manufacturing and post-utility management. Figure 1 presents system boundaries and the scope of the research. Both phases could have an undesirable impact on environment. Negative influence of wind power plants could affect many different components of our life. Climate change, formation of radioactive compounds, acidification, and eutrophication of soil are possible threats. The LCA method is a valuable tool, which could assess the environmental burden based not only on the scientific hypothesis but on the real data. This provides an opportunity for objective analysis, assessment, and above all it could help advance the techniques of eliminating the negative environmental impact of machines and objects. However, the LCA procedures do not include a wide scope of objects connected with wind power plants, e.g., operators, machines, technical appliances, and their mutual relations.

Usage of renewable energy sources is always connected with some depletion of natural resources. Manufacturing of each element, working system, or whole device requires not only raw materials but also energy, mainly produced with a use of conventional fuels. Despite this, renewable energy sources definitely reduce depletion of non-renewable resources [1,2,3].

Wind power plants are counted as an environmentally friendly source of energy. They fulfil basic assumptions of sustainable development. This type of energy production is associated with significantly lower CO_2_ emissions than in the case of conventional power plants.

Rationality is a trait of aware human activity, consisting of selecting the right methods that leads to the final goal [4]. There is a need for changing the way how the environmental resources are managed. Ecological and energetic analysis should be used to maintain balance between constant development and protection of the environment. LCA is one of the basic tools of such analysis, which includes whole life cycle of the objects. LCA refines the designing process and helps choosing more ecological solutions thanks to the fact that it takes into account both environmental and energetic aspects of production.

LCA method makes it possible to include almost all of the external factors into consideration during life cycle analysis of wind power plant. The structure made during the study makes it easy to identify dependencies between input and output elements. Such a foundation provides possibility to indicate the place and stage of life cycle that has the biggest impact on environment [4,5].

The usage of the LCA method is recommended by many current strategies and dispositions that promote the terms of sustainable development. Due the LCA method, comparison of completely different strategies is viable [6].

Ecological and energetic analysis are carried out mostly to assess the impact of the life cycle of object on the human health, environment quality, or to estimate natural resources depletion. LCA is a complex way of assessing influence of interaction between a product and its surroundings. The complexity of this method results from its interdisciplinary nature.

The implementation of recommendations developed on the basis of the results of the LCA analysis brings measurable benefits not only to companies, but also to the environment and thus to society as a whole. It makes it possible to indicate and make people aware of the correlation between human activities and their consequences for the environment.

A small number of experimental and analytical studies are devoted to expenditures characterizing the entire life cycle of a wind power plant [7,8,9]. Life cycle stages: from design, through production, to post-utilization of raw material, plastic, and material potentials (sometimes entire assemblies are suitable for further use, e.g., towers, gears) of a wind power plant are of great importance for the economy and the environment. It is worth undertaking comprehensive analytical and experimental tests to determine the outlays in the life cycle of a wind power plant. It should be determined how large are the environmental inputs in the form of energy used and greenhouse gas emissions at all stages of its life cycle [10,11,12,13].

In the world literature, one can find analyses mainly concerning the assessment of profitability, environmental benefits, and productivity of wind farms [14,15,16,17]; however, there is no comprehensive assessment focusing on environmental aspects of manufacturing construction elements of wind power plants.

Vargas et al. [17] made the environmental analysis of two two-megawatt wind turbines located in Mexico. The scope of the analysis included the stages of production, construction, and utilization of wind farms. As a result of the analysis, it was shown that the nacelle and tower potentially have the greatest negative impact on the environment. On the other hand, Wei-Wang Cheng and Yi-Heng TEAH [18] analyzed the environment of small wind turbines with a horizontal axis of rotation type (HAWT). The analysis focused on the assessment of the building components of the wind farm and the greenhouse gas (GHG) emissions throughout the life cycle. On this basis, it was proved that the production phase and the fabrication of the generator make the highest contribution to GHG emissions, whereas the phase of materials and the fabrication of generator are responsible for the most energy consumption. Lombardi et al. [19] researched two turbines with vertical axis Darrieus H-Rotor type with one- and three-kilowatt nominal power. In the study two methods were used, respectively, CML-IA and Thermo-Ecological Cost, in the whole life cycle. Additionally, the comparison of the ecological impact between turbines and Italian power grid was taken. The researchers took into consideration four life cycle phases: the production and manufacturing phase, operational and maintenance phase, after-life treatment, and transportation. The results showed that the highest environmental impact was achieved in the case of raw material production and manufacturing category. Between two analyzed turbines, the three-kilowatt one was characterized by lower values of environmental impact than the one kilowatt one. Both turbines achieved smaller values of impact per electricity unit than an Italian energy mix. Kouloumpis et al. [20] conducted an environmental analysis of a Darrieus five-kilowatt vertical axis wind turbine in terms of its efficiency. On this basis, it was proved that most of the environmental impacts are attributed to the infrastructure and not to specific elements of the wind farm construction. Alsaleh and Sattler [21] took LCA analysis for two-megawatt Gamesa wind turbines located in Texas, US. Modelling process was realized according to ISO 14040 standards [22] with use of SimaPro8 software. Raw material acquisition and manufacturing phases showed highest overall impact (higher than 60%) for all of the researched categories. 

Based on the above studies, it was not possible to directly compare the obtained research results with other researchers. The reason for this state of affairs is application of different test methods (e.g., CED—Cumulative Energy Demand or CML—Centrum voor Milieuwetenschappen), different geographical ranges (e.g., Mexico, Taiwan, Poland, USA), and different wind turbines. The conducted analytical and research procedure constitutes a valuable source of data for current and future constructions in terms of sustainable development of clean technologies, with particular emphasis on innovative wind farms.

The main objective of the work was the environmental analysis of the life cycle of an innovative wind power plant in terms of the resources used.

## 2. Materials and Methodology

### 2.1. Goal and Scope of the Analysis

The purpose of the environmental impact analysis was to identify potential negative environmental impacts. These influences occur at different material stages in the life cycle of objects. In this case, an innovative wind turbine with a capacity of 15 kW was analyzed. The structure consisted of a set of three turbines mounted on one supporting structure [23]. The environmental assessment took into consideration five group of elements of research unit: tower, turbine structure, rotor, generator, and instrumentation. The environmental impact was determined using the Eco-indicator 99 method. This method made it possible to assess the impact of the processes which take place within the life cycle of the power plant in eleven impact categories. Impact categories, specific to the Eco-indicator 99 model are: carcinogens, respiratory organics, respiratory inorganics, climate change, radiation, ozone layer, ecotoxicity, acidification/eutrophication, land use, minerals, and fossil fuels [24]. The scope of the research was related to European conditions, because life cycle stages of the research objects take place in Europe.

The aim of the conducted research was an implementation of an ecological assessment of the life cycle of an innovative wind power plant. Thanks to the undertaken research, it was possible to describe the existing reality (retrospective LCA) in terms of chemical compounds emitted into the natural environment. Simultaneously, it was possible to model future changes and implement more ecological, environmentally friendly solutions (prospective LCA). The LCA process was carried out in accordance with the ISO 14,000 family of standards guidelines [24]. The main research task was to determine the level of negative (or positive) impact of the life cycle of the tested object on human health, ecosystem quality, and depletion of natural resources [24,25].

ISO 14041 [26], in addition to ISO 14040 [22], sets out the requirements and procedures necessary to prepare and define the purpose and scope of a life cycle analysis, and to perform, interpret, and document an input and output set analysis (LCI).

### 2.2. Object and Plan of Analysis

**Figure 2:**
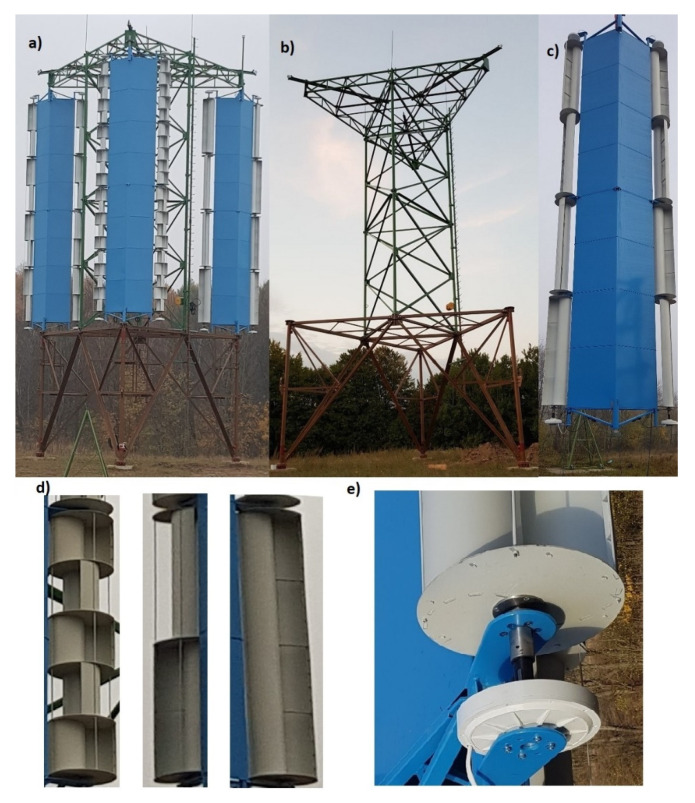
(**a**) Wind turbine; (**b**) wind turbine tower; (**c**) wind turbine structure; (**d**) rotors 1-, 2-, 5-moduls; (**e**) generator.

An innovative 15 kW wind power plant was subject of research. The total height of the structure was 15.5 m and the working space was between 5 and 14.5 m above ground level (Figure 2a). The wind power plant consisted of three 5-kW windmills, with dimensions 9.5 m high and 2.8 m wide placed on one tower (Figure 2b).

Each turbine contained two rotor columns divided in half into two sections. One single section drives one generator. That way an optimization research of power generation efficiency for local wind conditions could be taken. Such a solution makes it necessary to manage twelve sources of energy in a whole set of turbines, which work independently of each other.

An important feature, however, not modeled in this publication, is that thanks to the installed equipment, it is possible to demount the turbines and service them without using a crane or a basket lift. This feature is almost unique in other wind turbine constructions and will have a positive impact on reducing the environmental load during the exploitation of the wind turbine.

The turbine itself includes a turbine structure (Figure 2c), rotors (Figure 2d) and generators (Figure 2e). The structure under discussion is a prototype solution with a research purpose. Tests and experiments will be carried out for three different rotors mounted on the facility under scrutiny. The final construction will have one solution based on cost-environmental optimization. The prototype structure is in the vast majority made of steel (98% of its weight consist of easy to recover steel), which makes it possible to reuse it in metallurgical processes. Only generators and electronic equipment will be able but not easily to be reused.

The conclusions contained in this publication can be considered and applied in the commercial version of the turbine. The main method of evaluating the potential environmental impact of a wind turbine, on human health, ecosystem quality, and depletion of raw materials resources was a Life Cycle Analysis. The LCA analysis performed in this work included four stages (according to ISO 14000 family of standards): determination of goal and scope, life cycle inventory (LCI), life cycle impact assessment (LCIA), and interpretation [25].

In the beginning, the goal and research scope were formulated. Goal was defined by thorough analysis of the state-of-the-art research. It was been noticed that the literature lacks detailed environmental assessments of innovative low-capacity wind farms. Wind power plants are designed to meet the highest national and European standards and with special care for environment. The key role in goal and research scope formulation was played by comprehensive data collection. It was possible thanks to the cooperation with scientists from the Polish Academy of Sciences [23,25]. The next step included the detailed life cycle assessment of the whole building process of the innovative wind turbine power plant. The simulations were carried out with a use of SimaPro 8.4.0 software (Pré Consultants B.V., Amersfoort, The Netherlands). Calculations were based on the Eco-indicator 99 method. This method created the possibility of assessing all life cycle processes in the eleven impact categories. The complete course of the third stage with all of the above-mentioned results (with detailed overview) and research results interpretation [27,28] is presented in Section 3.

### 2.3. System Boundary and Functional Unit

**Figure 3:**
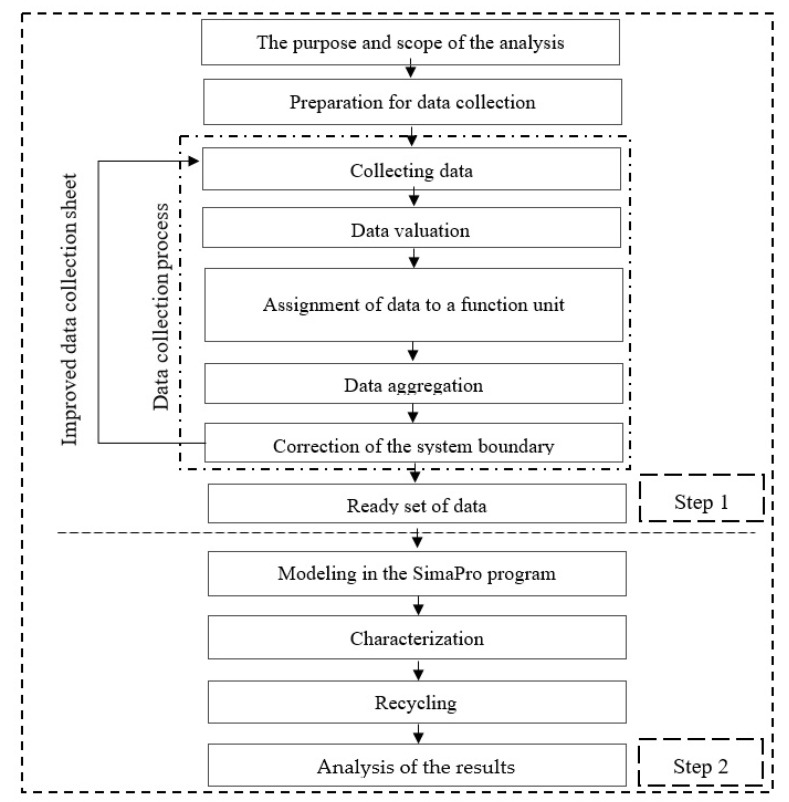
Detailed flow diagram of used methodology.

Analysis was performed with assumption that manufacturers and the wind power plant are located in Poland. Electricity production was a function of the facility. Productivity of the wind power plant at the stage of its production was assumed as the functional unit. The analysis assumes a 25-year life cycle of the power plant. The stages of sales, technical tests, and storage were excluded from the system, which is due to lack of relevant data. The flowchart below (Figure 3) shows the sequence of operations undertaken in our research. There are two main steps. The first one is to acquire a database whose architecture and acquisition plan determines the subsequent calculation possibilities. The second step is to model and carry out research using the SimaPro program (Pré Consultants B.V.) and end with analysis and conclusions at the level of characterization. Further stages of the analysis are described in Article 2/2.

### 2.4. Life Cycle Inventory (LCI)

Life Cycle Inventory consists of creating an input-output set. Therefore, it is a balance sheet analysis based on a data inventory [29]. In order to gather the data required for the analysis with the greatest possible precision, special inventory sheets were constructed. An individual sheet was assigned to each individual process. Within the sheets, inputs and outputs from the process were specified and data related to its implementation were taken into account. Inputs included main and Supplementary Materials [30,31]. Outputs included the main product in the form of a turbine and emissions. The data was related to the prototype solution of the wind power plant. The input data for the materials and components of the wind power plant was part of own-research. In order to create an inventory table, individual environmental impacts of the same type were summed up for all individual processes [32]. 

Table 1 contains a list of materials and elements constituting the core of the life cycle analysis for a wind power plant over a 25-year lifetime. 

Once all of the data had been allocated to the individual processes the validation process by means of a bilateral energy and mass balance was conducted [33]. The models were systematically built and filled with data. The volume of inputs was equal to the volume of outputs [34]. This procedure allowed data aggregation, conversion into functional units, and reference streams. Input-output matrices were created as the result of summation of the same type of data (inputs of materials, energy, emissions, etc.) for individual unit processes. In the next step, the matrices were assigned to reference streams, resulting in inventory tables [35,36]. All of the data had to be adapted to the SimaPro 8.4.0 software (Pré Consultants B.V.) format. After entering data into the software, it was possible to move on to the third research stage—LCIA. 

### 2.5. Life Cycle Impact Assessment (LCIA) Eco-Indicator 99 Method

**Figure 4:**
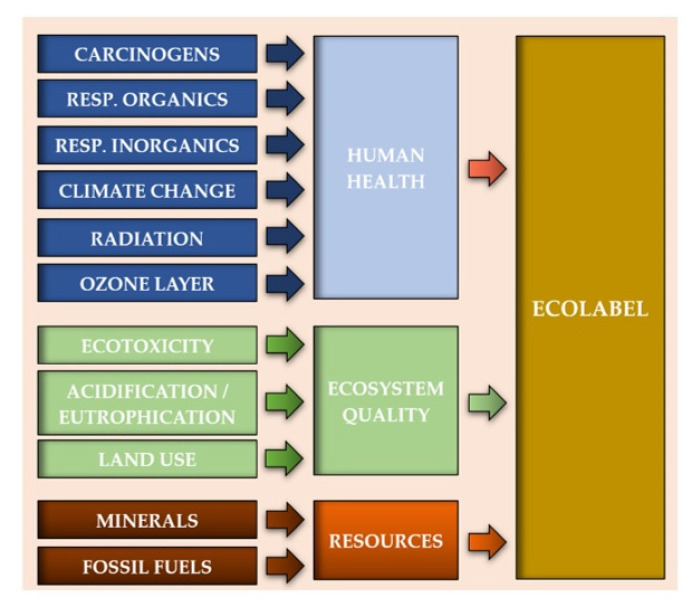
The method of data aggregation in the Eco-indicator 99 method.

The Eco-indicator 99 method is based on the modelling of the environmental impact at the environmental mechanism endpoint level. All standard impact categories were included in the analysis using this model: carcinogens, respiratory organics, respiratory inorganics, climate change, radiation, ozone layer, ecotoxicity, acidification/eutrophication, land use, minerals, and fossil fuels [37]. Their selection was consistent with the purpose and scope of the research. These impact categories can be grouped into three larger groups, defined as areas of influence: human health, ecosystem quality, and resources. Areas of influence can be summed up in the form of the final Ecolabel after performing standardization, grouping, and weighting (Figure 4) [29,38,39].

Once the impact category was established and selected, the classification began. Classification included assigning the LCI results to each impact category. Thanks to this procedure, it was possible to conduct characterization, which consists of calculating the value of the category index for LCI results (using the parameter of characterization). This makes it possible to assess the level of their contribution to the size referring to a given impact category. The result is the numerical value of the indicator. Climate change category may serve as an example in which carbon dioxide and methane emissions play key roles. The final result is the value of the indicator expressed as an equivalent of carbon dioxide, e.g., kg CO_2_ eq. Similarly, index values are set for the other impact categories [25,33,40,41].

In the Eco-indicator 99 method, indicators are taken from a subsequent level of the environmental mechanism. In the case of human health, the DALY (Disability Adjusted Life-Years) unit is used, and as a category indicator—YLL (Years of Life Lost) and YLD (Years Lived Disabled) units. DALY is an internationally recognized unit, used by the WHO and the World Bank to evaluate health statistics. Various diseases are assigned to the DALY scale where 0 is ideal health and 1 means death. The DALY unit expresses six impact categories: carcinogens, respiratory organics, respiratory inorganics, climate change, radiation and ozone layer [28,31,42].

Influences responsible for decrease in ecosystem quality are more varied compared to the area of human health assessment. There is no common unit for such impacts. In the Eco-indicator 99 method, the indicator represents the level of species diversity. The unit is PAF (Potentially Affected Fraction) or PDF (Potentially Disappeared Fraction). Representative species were selected within three impact categories assessed in this area (ecotoxicity, acidification/eutrophication, land use). For ecotoxicity (PAF-m^2^/yr), these were lower terrestrial and aquatic animal species, while acidification/eutrophication and land use (PDF-m^2^/yr) were referred to selected vascular plant species. It is also possible to convert the PAF unit to PDF [28,43,44]. 

Modelling of resources consists of resources and damage analysis. For this purpose, a special damage indicator was developed—surplus energy express in MJ. The possible effects of extraction processes are decrease in the useful component of the deposit or its total extraction. In the case of supply reduction (partial or total depletion of the deposit), it will become necessary to provide additional energy to extract this resource in the future [25,45]. Therefore, if the quality of a certain resource decreases (as a result of increased extraction), the effort to extract it from other sources increases accordingly. The use of one kilogram of a given resource is associated, on the one hand, with a reduction in its quality and, on the other, with an increase in the effort to extract it (surplus energy). In Eco-indicator 99, two impact categories are expressed in MJ surplus energy: minerals and fossil fuels [46,47]. 

## 3. Results

The results of the Life Cycle Impact Assessment (LCIA) analyses included damage and impact categories of the Eco-indicator 99 method [48,49]. The results of modelling using Eco-indicator 99 were divided into eleven damage categories and three impact categories [50]. All results are presented in units specific to the characterization step. The study using Eco-indicator 99 analyzed in detail eleven impact categories that are specific to this model: carcinogens, respiratory organics, respiratory inorganics, climate change, radiation, ozone layer, ecotoxicity, acidification/eutrophication, land use, minerals, and fossil fuels [50,51].

The results were collated for the life cycle of the innovative wind power plant at the manufacturing stage and additionally compared with the recycling model. The first step included identification of the most influencing category in terms of negative (or positive) environmental impact during the life cycle, then results of analysis were compared for two cases: with or without recycling [50,52].

**Figure 5:**
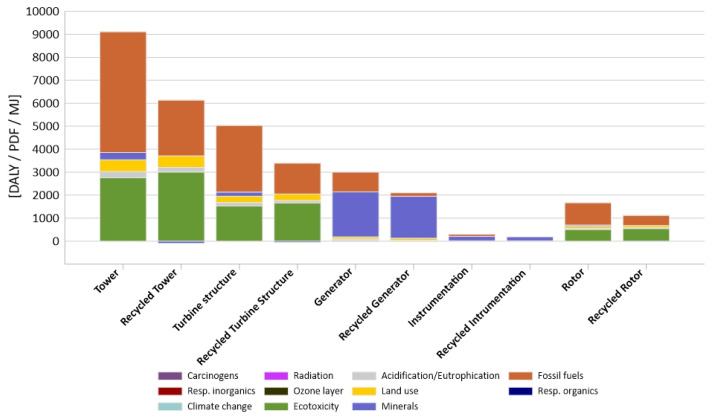
Results of characterization of environmental impact occurring during the life cycle of an innovative wind power plant elements, comparison of results with and without recycling.

Figure 5 shows comparative results of Table 2 and Table 3. This graph should be only considered as a guideline, as values with different units are presented on one axis. However, this representation enables a visual comparison of the changes of individual pairs of elements with and without recycling as well as pairs between each other. Similarities can be seen in the tower and turbine structure as the differences from the generators, both in terms of the structure and their recyclability. Among the factors that may have a negative impact on human health, the highest level of harmful impacts was characterized by a group of inorganic compounds causing respiratory diseases (from 0.00019 DALY for the equipment to 0.0063 DALY for the tower). In the group of factors affecting the decrease in the ecosystem quality, the category of ecotoxicity was of key importance (from 3.6 PDF-m^2^/yr for the instrumentation to 2759 PDF-m^2^/yr for the tower). In the midst of the factors associated with the depletion of fossil resources, by far the most harmful impact were the fossil fuel extraction processes (from instrumentation 89 MJ, 858 MJ generator, 963 MJ rotor, 2908 MJ turbine structure to 5257 MJ for the tower). Due to the high energy demand during production and the related energy-consuming processes of extracting non-renewable resources.

Modelling, which took into consideration recycling (Table 3 and Figure 5), showed that the group of inorganic compounds causing respiratory diseases had the most negative impact on human health (from 0.00012 DALY for instrumentation to 0.0045 DALY for tower). In the group of factors affecting the deterioration of the quality of the environment it is a category of ecotoxicity (from −8.2 PDF-m^2^/yr for instrumentation to 3005 PDF-m^2^/yr for the tower). Among the factors related to the depletion of fossil resources, the processes of fossil fuel extraction had the greatest influence (from instrumentation −19 MJ, through 143 MJ generator, 444 MJ rotors, 1342 MJ turbine structure, to 2425 MJ for the tower).

Almost all of the chemicals that accompany processes in the life cycle of wind turbines are possible threats for environment. Therefore, in environmental and energy analyses, for the determination of potential risks, a substance is assumed to have an impact on each of the impact categories to which it may potentially contribute [24,41,52].

The two components of the wind turbine, the tower and the turbine structure are very similar in terms of material composition (99.9% is steel) and high weight (6678 kg tower, 3454 kg turbine structure) to the other components (Figure 5).

Table 4 presents the results of characterization of the environmental impact of the wind turbine tower elements. The research carried out revealed particularly high level of negative impacts of profile steel. For which, in the group of factors influencing the reduction of environmental quality, the highest potential impact was observed for the ecotoxicity category 2524 PDF-m^2^/yr. The processes of extraction of fossil fuels had the second highest level of potentially harmful environment changes (4811 MJ).

The reuse of materials originally used in the production of an innovative wind turbine could become a potential source of environmental and health benefits. This is particularly visible in the area of reducing atmospheric emissions of chemical compounds released during the extraction of fossil fuels. Recycling used in the production of steel allows to reduce the level of potential environmental impact to 2425 MJ from 5257 MJ, in the perspective of their entire life cycle. 

Ecotoxicity is a parameter of some substances, which may cause an immediate (or delayed) risk to one (or more) environmental compartments. Such substances may therefore have a significant impact on the quality of the environment and, consequently, be a threat to human and animal health. From all of the studied components during the manufacturing of the turbine structure, the highest level of emissions of ecotoxic compounds was recorded for the profile steel (18G2A—S355JR) from which the turbine structure was made (Table 5). Wind turbine production requires a lot of energy and materials. Exceptionally high levels of potential negative impacts were recorded for the fossil fuels category, where the use of steel was responsible for an impact level of 2785 MJ. The impact category characterized by the highest level of potential harmful effects on human health was the emission of inorganic compounds causing respiratory diseases. The application of recycling processes to the analyzed elements of the wind turbine may significantly reduce the potentially negative impact on the environment in the perspective of their entire life cycle. The highest level of reduction was observed for fossil fuels (−1566 MJ) and for minerals (−231 MJ).

Exploitation of minerals causes not only the depletion of resources, but also the violation of the current state of the environment, which is reflected in its transformation and deterioration. In the case of extraction of raw materials, changes in the environment significantly depend, on methods of underground, open-pit, or borehole extraction. In the case of an innovative wind turbine, the most potential negative environmental effects associated with the extraction of mineral resources are distinguished by the life cycle of the generator 1958 MJ Table 6, while the least—the life cycle of instrumentation 187 MJ. For the fossil fuels category for the instrumentation element, a positive environmental impact was observed, as the environmental benefit was equal to −109 MJ and the impact level at the manufacturing stage was only 89 MJ. The use of recycling methods can reduce (to a greater or lesser extent) the level of negative impacts on human health over their entire life cycle. The highest level of reduction was observed for the generator in the Carcinogens category −0.000059 DALY. 

One of the tasks of the 3-Pio-Wiat project was to determine which of the three rotor designs is more economical and efficient. Therefore, an environmental analysis was also carried out for each of them separately (Table 7).

The highest potential level of negative influences was characterized by the rotor concept with five modules. In the case of the fossil fuel category, potentially the highest emission level of 364 MJ was registered for rotor 5, slightly less for rotor 2, as much as 307 MJ, and the lowest emission level for rotor 1, that is 292 MJ. Recycling reduces the depletion of non-renewable resources, but also significantly reduces environmental degradation. Rotor recycling processes make it possible to reduce the level of potential harmful effects of processes related to the extraction of fossil fuels to the greatest extent. In the perspective of the entire life cycle of the analyzed research object (to the level below 50%). Recycling may result not only in the complete elimination of harmful effects in the perspective of the life cycle of the turbine, but even in an increase of the level of environmental quality, as may be the case with the minerals and carcinogens category.

**Figure 6:**
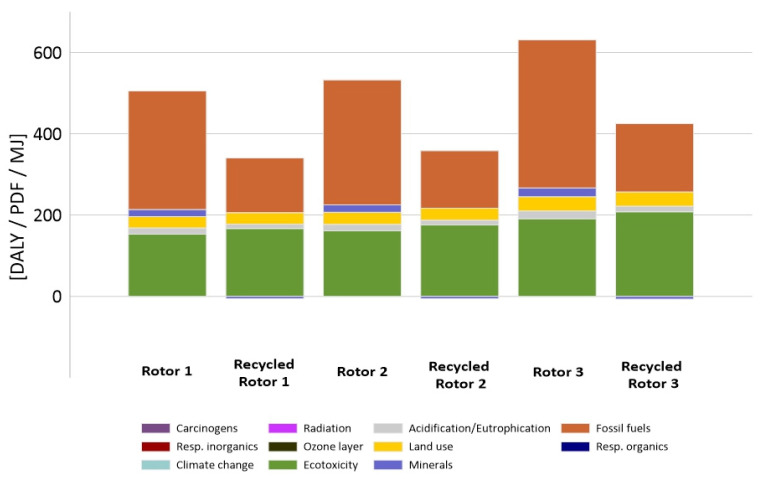
Results of characterization of the environmental impact during the rotors life cycle of the model with and without recycling. One-, two-, and five-segment rotors.

The differences in environmental impact between the different rotor solutions can be seen in Figure 6. This trend remains true for both the non-recycling and the recycling models.

**Figure 7:**
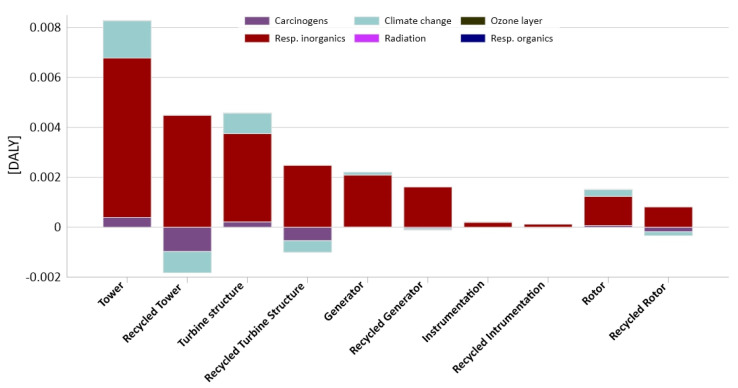
Results of characterization of environmental impacts occurring during the life cycle of elements of the construction of an innovative wind power plant comparison of results with and without recycling for the human health categories.

**Figure 8:**
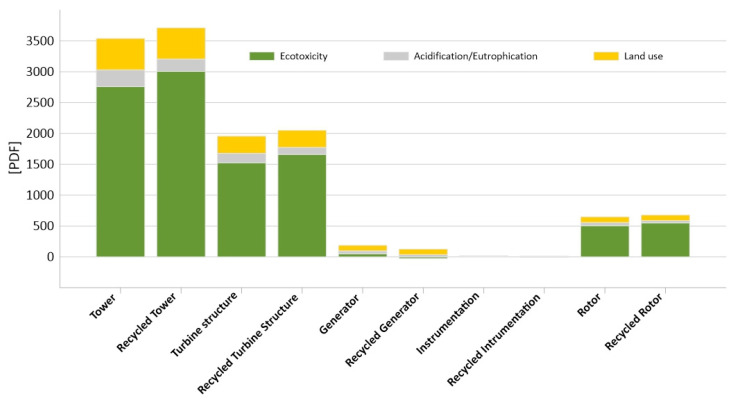
Results of characterization of environmental impacts occurring during the life cycle of elements of the construction of an innovative wind power plant comparison of results with and without recycling for the ecosystem quality categories.

**Figure 9:**
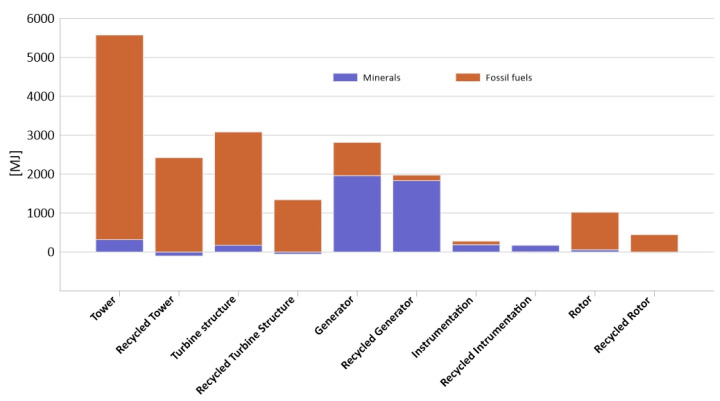
Results of characterization of environmental impacts occurring during the life cycle of elements of the construction of an innovative wind power plant comparison of results with and without recycling for the resources categories.

As can be seen in the tables above, addition of the recycling element to the analysis had a different impact on the final result depending on the impact categories. For the clarity, the results of each impact category are placed on a separate charts: Figure 7 (human health), Figure 8 (ecosystem quality), Figure 9 (resources). The negative values obtained were a constant element of the method caused by different environmental impacts of materials in the primal and secondary cycle and were not treated as analysis errors [53]. 

In the human health group (Figure 7), we can clearly notice a reduction in negative impacts after applying the recycling method, when in the ecosystem quality (Figure 8), indicators even become worse. However, the most beneficial impact is seen in the resources category (Figure 9), which is clearly dependent on the materials of which the object is composed.

## 4. Summary and Conclusions

The pro-ecological improvements for products are achieved mainly through rationalization of resources usage and measures to reduce the amount of waste and pollution. Rationalization of the exploitation of natural resources consists of reducing the material and energy consumption by processes throughout all phases of the product life cycle. The research analyzed two phases in the life cycle of an innovative wind turbine: manufacturing and recycling. The possibility of reducing the consumption of resources in the production phase is ensured by an appropriate selection of materials and manufacturing technology. Currently, it is recommended to use technologies with low material and heat loss and, if possible, to implement a technology of metal forming. The development and implementation of waste-free technologies is very difficult due to the need for different physical and chemical processes, which are often very expensive. All products that are withdrawn from use after the end of the life of the wind turbine are potential objects of recycling. If it is not possible to use post-consumer waste as secondary raw materials for the production of new wind turbines, it can be used to create other products.

The usage of the LCA method could provide very tangible benefit for companies that want to reduce their negative impact on the natural environment. Ecological life cycle assessment is a tool that supports decision making on environmental protection, for the people responsible for it. Thanks to the results of the LCA method, the producer is able to find the optimal solution to minimize the environmental impact of the existing process. All attempts to describe the world and the impact of human activities on the whole surrounding must be confronted with the application of certain restrictions and universalization. As such, LCA analyses must be subject to this principle, and although methods such as Eco-indicator 99 are constantly being improved and modified, they will never reach full excellence. 

The LCA allows not only to analyze the whole object, but also to look at individual phases of the cycle of existence, either as a single component or as a group of components. Life cycle assessment is performed for an existing specific product, and in the case of complex products, it can be based on a specific production stage. On the basis of the applied system boundaries, it is possible to make a comparison of the investigated phases of the life cycle. This analysis enables to determine the effects that a new product or process may have on the environment. Properly verified results of analyses made during design phase allow to eliminate future negative environmental effects. These actions will certainly help many manufacturers around the world to invest deliberately in solutions or modernization of production processes in order to achieve clean production.

The aim of the work was achieved by developing a methodology for evaluating the ecological and energy efficiency of an innovative wind turbine using LCA methodology. The conducted study was based on the Eco-indicator 99 method based on the environmental endpoint mechanism methodology. The results of the research were provided for five components of a complete wind turbine: the tower, turbine structure, rotors, generators, and instrumentation.

The analysis of environmental costs at the stage of production of the innovative wind power plant showed that the highest environmental costs in the form of negative impact on human health were noted in the case of the tower (total: 0.0083 DALY), the lowest in the case of instrumentation (total: 0.0002 DALY). Vargas et al. [17] achieved similar results in the case of their 2.0-MW turbines. The highest impact was shown for nacelle and tower. On the other hand Wang and Teah [18] showed that in the case of small-scale HAWT turbine, the generator had the biggest environmental impact. In their study, vast majority of turbine weight was from the generator. Such comparison shows that it is hard to collate LCA studies for different wind turbines. Each wind turbine could be of different type or size, which strongly influences achieved results. Even between exactly the same turbines placed on different heights (e.g., one on the top of the building and another one on high tower), results could vary. Comparison between studies in terms of the weighted environmental outcome or in terms of recycling influence seems to give more accurate conclusions. The emission of inorganic compounds causing respiratory diseases was a key contributor to the total amount of potential negative impact in this category. 

The highest potential negative environmental impact, in the case of all the examined elements of wind turbine construction, was identified for categories related to fossil fuel extraction (total: 10,076 MJ). Such a result correlates with different studies [17,20], which also showed that abiotic depletion in the case of fossils had the biggest impact on the environment. Mining and processing of metals and fossil fuels are the cause of the greatest changes in the environment. Their functioning consists in obtaining various raw materials, and from the point of view of the environmental burden, the most important are the quantities of raw materials extracted and the methods of their extraction.

Of all of the considered emission areas, atmospheric emissions represent the largest share for the Ecotoxicity impact category (total: 4844 PAF-m^2^/r). The production processes are usually related to the consumption of energy obtained from conventional sources, which increases the level of potential negative impact on the life cycle environment. Among the five main elements of wind turbine construction, the greatest environmental damage was observed for the tower (2759 PAF-m^2^/r), while the least environmental damage was caused by the instrumentation (3.6 PAF-m^2^/r).

The economic use of waste as secondary raw materials from exploited parts of wind turbines is defined as recycling. The possibility of reusing them is due to the fact that they are made of renewable materials. The use of recycling processes can reduce the level of negative impact in the perspective of the entire life cycle of a wind turbine, typically by about 30%. It is estimated that steel recycling saves up to 72% of the energy needed for primary production, and recycling of one ton of steel saves 1.4 tons of iron ore, 0.8 tons of coal, 0.3 tons of limestone, and 1.67 tons of CO2. On the other hand, using recycled steel for the production of new steel reduces air pollution by about 86%, water consumption by about 40%, and water pollution by 76% [54]. 

Among all the available data, it is worth paying particular attention to the environmental benefits resulting from the recycling processes used. In the case of the environmental assessment of the instrumentation, we obtain an environmental benefit of approximately 20%. For the fossil fuels category, for example, the environmental impact of a tower in a non-recycling model has an impact of 5258 MJ and a recycling model has an impact of 2322 MJ, which is a 56% reduction. Such great results were obtained thanks to the unique design of wind turbine under consideration. Not all types of wind turbines are meant to after-life treatment, e.g., in the case of the small-scale HAWT turbine [18], recycling provides only small impact on environment (around 0.2%) and it could be economically unprofitable.

In the construction of innovative wind power plants, there are many opportunities to design the object in such a way that leads to reducing the consumption of natural resources and reduce the burden on the environment. First of all by reducing natural energy resources and water usage. Implementation of more efficient designs is one of the main principles of optimization. Studied wind turbine is characterized by higher amount of materials used in the manufacturing processes, however components of this turbine are easily recyclable, which leads to huge environmental gains. Another principle that should be taken into account during the designing phase are maintenance and modernization of the wind turbine. Construction that enables simplified operation for working teams could also reduce unnecessary actions and as a result reduce environmental impact. The energy consumption is affected both by the way in which the turbine and its installations are operated and by the operation of equipment ensuring its uninterrupted functioning. The principle of optimization of manufacturing processes results in a goal of minimizing the amount of natural resources used for the production of technical objects. 

## Figures and Tables

**Table 1:** Summary of materials and components of a 15 kW wind power plant.

Component	Element	Material	Total Mass (kg)	Al (kg)	Cu (kg)	Steel (kg)	Plastics/Kind (kg)	Neodymium (kg)	Other (kg)
Tower									
Profile steel	Pipe	S365JR	968.86	-	-	968.86	-	-	-
	Plate	S365JR	1068.02	-	-	1068.02	-	-	-
	Angle iron	S365JR	3258.23	-	-	3258.23	-	-	-
	Square profile	S365JR	776.70	-	-	776.70	-	-	-
	Welds	S365JR	39.38	-	-	39.38	-	-	-
Total profile steel		-	6111.19	0	0	6111.19	0	0	0
Tower connectors	Bolts	S365JR	78.06	-	-	78.06	-	-	-
	Washers	S365JR	7.59	-	-	7.59	-	-	-
	Nuts	S365JR	47.75	-	-	47.75	-	-	-
	Platform gratings	S365JR	81.21	-	-	81.21	-	-	-
Total connectors		-	214.61	0	0	214.61	0	0	0
Turbine service mechanism	Crane	S365JR	282.45	-	-	282.45	-	-	-
	Welds	S365JR	1.50	-	-	1.50	-	-	-
	Winch mounting	S365JR	6.50	-	-	6.50	-	-	-
	Winch	mix	62.00	-	-	62.00	0.20	-	-
Total turbine service mechanism			352.65	0	0	352.45	0.20	0	0
Total Tower		-	6678.45	0	0	6678.25	0.20	0	0
Turbine structure									
Profile steel	Plate	S365JR	1264.62	-	-	1264.62	-	-	-
	Pipe	S365JR	986.10	-	-	986.10	-	-	-
	Square profile	S365JR	301.59	-	-	301.59	-	-	-
	Rectangle Profile	S365JR	717.51	-	-	717.51	-	-	-
	Welds	S365JR	29.58	-	-	29.58	-	-	-
Total profile steel		-	3299.40	0	0	3299.40	0	0	0
Turbine structure connectors	Bolts	S365JR	6.96	-	-	6.96	-	-	-
	Washers	S365JR	0.54	-	-	0.54	-	-	-
	Nuts	S365JR	4.20	-	-	4.20	-	-	-
Total connectors	-	-	11.70	0	0	11.70	0	0	0
Bearing-coupling system	Rotor bearings	Mix	12.51	-	-	12.48	0.03	-	-
	Turbine bearings	Mix	72.06	-	-	72.00	0.06	-	-
	Bearing mountings	S365JR	44.64	-	-	44.64	-	-	-
	Coupling system	Steel	15.48	-	-	15.48	-	-	-
Total bearing-coupling system		-	144.69	0	0	144.60	0.09	0	0
Total turbine structure		-	3455.79	0	0	3455.70	0.09	0	0
Rotors									
Rotor 1	Plate	S365JR	326.32	-	-	326.32	-	-	-
	Pipe	S365JR	43.28	-	-	43.28	-	-	-
	Welds	Steel	0.80	-	-	0.80	-	-	-
Total rotor 1		-	370.40	0	0	370.40	0	0	0
Rotor 2	Plate	S365JR	345.44	-	-	345.44	-	-	-
	Pipe	S365JR	43.28	-	-	43.28	-	-	-
	Welds	Steel	1.60	-	-	1.60	-	-	-
Total rotor 2		-	390.32	0	0	390.32	0	0	0
Rotor 3	Plate	S365JR	415.36	-	-	415.36	-	-	-
	Pipe	S365JR	43.28	-	-	43.28	-	-	-
	Welds	Steel	4.00	-	-	4.00	-	-	-
Total rotor 3		-	462.64	0	0	462.64	0	0	0
Total rotors		-	1223.36	0	0	1223.36	0	0	0
Generators									
Generator	Generator	mix	264.00	65.64	48.00	104.76	4.80 polypropylene (PP)	36.00	-
							4.80 polyethylene (PE)		
Total generator		-	264.00	65.64	48.00	104.76	9.60	36.00	0
Instrumentation									
Wiring	Turbine wiring (3 × 1.5 26.16 m)	mix	2.36	-	0.92	-	1.44 (PVC)	-	-
	Tower wiring (3 × 1.5 29.62 m)	mix	2.67	-	1.04	-	1.63 (PVC)	-	-
Turbine control module	Turbine control module	mix	20.40	10.00	2.40	6.00	-	-	2.00
Commutator	Commutator	mix	1.80	0.30	0.30	0.15	1.05 (PE)	-	-
Total instrumentation		-	27.23	10.30	4.66	6.15	4.12	0	2.00
Total wind turbine		-	11648.83	75.94	52.66	11468.22	14.01	36.00	2.00

**Table 2:** Results of characterization of environmental consequences occurring in particular stages of the life cycle of an innovative 15 kW wind power plant, including categories of impacts without recycling.

Impact Categories	Unit	Tower	Turbine Structure	Rotor	Generator	Instrumentation
Carcinogens	DALY	0.0004	0.0002	0.000072	0.0000064	0.00000038
Respiratory organics	DALY	0.00001	0.0000057	0.0000019	0.0000007	0.00000014
Respiratory inorganics	DALY	0.0063	0.0035	0.0012	0.0021	0.00019
Climate change	DALY	0.0015	0.00083	0.00027	0.00012	0.000012
Radiation	DALY	0	0	0	0	0
Ozone layer	DALY	0	0	0	0	0
Ecotoxicity	PAF × m^2^ yr	2759	1525	505	51	3.6
Acidification/Eutrophication	PDF × m^2^ yr	273	150	50	48	4.4
Land use	PDF × m^2^ yr	506	280	93	89	10
Minerals	MJ	315	174	58	1958	187
Fossil fuels	MJ	5257	2907	963	858	89

**Table 3:** Results of characterization of environmental consequences occurring in particular stages of the life cycle of an innovative 15 kW wind power plant, including categories of impacts with recycling.

Impact Categories	Unit	Recycled Tower	Recycled Turbine Structure	Recycled Rotor	Recycled Generator	Recycled Instrumentation
Carcinogens	DALY	−0.00097	−0.0005	−0.00018	−0.000053	−0.0000066
Respiratory organics	DALY	0.0000058	0.0000032	0.0000011	−0.0000003	−0.00000005
Respiratory inorganics	DALY	0.0045	0.0025	0.00082	0.0016	0.00012
Climate change	DALY	−0.00085	−0.00047	−0.00016	−0.00006	−0.000011
Radiation	DALY	0	0	0	0	0
Ozone layer	DALY	−0.000001	−0.0000006	−0.0000002	−0.0000003	−0.000000038
Ecotoxicity	PAF × m^2^ yr	3005	1661	550	−27	−8.2
Acidification/Eutrophication	PDF × m^2^ yr	200	110	37	37	2.8
Land use	PDF × m^2^ yr	506	280	93	89	10
Minerals	MJ	−102	−57	−19	1830	168
Fossil fuels	MJ	2425	1341	444	143	−19

**Table 4:** Results of characterization of environmental impacts at the stage of production and recycling of an innovative wind turbine tower.

Impact Category	Unit	Profile Steel	Tower Connectors	Turbine Service Mechanism	Tower Total	Recycling	Tower Total Recycled
Carcinogens	DALY	0.00036	0.000013	0.000021	0.0004	−0.00137	−0.00097
Respiratory organics	DALY	0.0000095	0.00000003	0.0000005	0.00001	0.0000046	0.0000058
Respiratory inorganics	DALY	0.0058	0.00021	0.00034	0.0063	−0.0019	0.0045
Climate change	DALY	0.0014	0.000048	0.000079	0.0015	−0.0024	−0.00085
Radiation	DALY	0	0	0	0	0	0
Ozone layer	DALY	0	0	0	0	−0.000001	−0.000001
Ecotoxicity	PAF × m^2^ yr	2524	89	146	2759	246	3005
Acidification/Eutrophication	PDF × m^2^ yr	250	8.8	14	273	−73	200
Land use	PDF × m^2^ yr	463	16	27	506	0	506
Minerals	MJ surplus	288	10	17	315	−418	−102
Fossil fuels	MJ surplus	4811	169	278	5257	−2833	2425

**Table 5:** Results of characterization of environmental impacts at the manufacturing and recycling stage of a turbine structure of an innovative wind turbine.

Impact Category	Unit	Profile Steel	Turbine Structure Connectors	Bearing, Coupling System	Turbine Structure Total	Recycling	Turbine Structure Recycled
Carcinogens	DALY	0.00021	0.0000006	0.0000086	0.0002	−0.00076	−0.0005
Respiratory organics	DALY	0.0000055	0.0000000	0.00000023	0.0000057	−0.0000025	0.0000032
Respiratory inorganics	DALY	0.0034	0.00001	0.00014	0.0035	−0.001	0.0025
Climate change	DALY	0.0008	0.0000024	0.000033	0.00083	−0.0013	−0.00047
Radiation	DALY	0	0	0	0	0	0
Ozone layer	DALY	0.00000001	0	0	0	−0.00000061	−0.0000006
Ecotoxicity	PAF × m^2^ yr	1461	4.34	60	1525	136	1661
Acidification/Eutrophication	PDF × m^2^ yr	145	0.43	5.9	150	−40	110
Land use	PDF × m^2^ yr	268	0.8	11	280	0	280
Minerals	MJ surplus	167	0.5	6.8	174	−231	−57
Fossil fuels	MJ surplus	2785	8.2	115	2907	−1566	1341

**Table 6:** Results of characterization of environmental consequences at the stage of manufacturing and recycling of instrumentation and generators of an innovative wind turbine.

Impact Category	Unit	Generator	Recycling	Generator Recycled	Instrument-Ation	Recycling	Instrumentation Recycled
Carcinogens	DALY	0.0000064	−0.0000593	−0.000053	0.00000038	−0.000007	−0.0000066
Respiratory organics	DALY	0.0000007	−0.0000011	−0.0000003	0.00000014	−0.0000002	0
Respiratory inorganics	DALY	0.0021	−0.00047	0.0016	0.0002	−0.000071	0.00012
Climate change	DALY	0.00012	−0.00018	−0.000059	0.000012	−0.000024	−0.000012
Radiation	DALY	0	0	0	0	0	0
Ozone layer	DALY	0	−0.0000003	−0.0000003	0	0	0
Ecotoxicity	PAF × m^2^ yr	51	−78	−27	3.6	−12	−8.2
Acidification/Eutrophication	PDF × m^2^ yr	48	−11	37	4.4	−1.7	2.8
Land use	PDF × m^2^ yr	89	0	89	10	0	10
Minerals	MJ surplus	1958	−128	1830	187	−19	168
Fossil fuels	MJ surplus	858	−715	143	89	−109	−19

**Table 7:** Results of characterization of environmental impacts at the manufacturing and recycling stage of the rotors of an innovative wind turbine.

Impact Category	Unit	Rotor 1	Rotor 1 Recycled	Roto 2	Rotor 2 Recycled	Rotor 5	Rotor 5 Recycled
Carcinogens	DALY	0.000022	−0.000054	0.000023	−0.000057	0.000027	−0.000067
Respiratory organics	DALY	0.0000006	0.0000003	0.0000006	0.0000003	0.0000007	0.0000004
Respiratory inorganics	DALY	0.00035	0.00025	0.00037	0.00026	0.00044	0.00031
Climate change	DALY	0.000083	−0.000047	0.000088	−0.00005	0.0001	−0.000059
Radiation	DALY	0	0	0	0	0	0
Ozone layer	DALY	0	−0.0000001	0	−0.0000001	0	−0.0000001
Ecotoxicity	PAF × m^2^ yr	153	167	161	176	191	208
Acidification/Eutrophication	PDF × m^2^ yr	15	11	16	12	19	14
Land use	PDF × m^2^ yr	28	28	30	30	35	35
Minerals	MJ surplus	17	−5.7	18	−6	22	−7.1
Fossil fuels	MJ surplus	292	135	307	142	364	168

## Data Availability

The data in the study are available on request from the corresponding author. The data are not publicly available due to privacy policies.
